# Prediction models for aspiration risk in stroke patients: a systematic review

**DOI:** 10.3389/fneur.2026.1700285

**Published:** 2026-04-16

**Authors:** Xiang-Ru Li, Hong Zhou, Fang-Ju Mao

**Affiliations:** 1 The First Affiliated Hospital of Yangtze University, Jingzhou, China; 2 Health Science Center, Yangtze University, Jingzhou, China

**Keywords:** aspiration, aspiration pneumonia, prediction model, stroke, systematic review

## Abstract

**Background:**

Stroke is a leading cause of disability and death, with post-stroke dysphagia significantly increasing aspiration risk, leading to complications, such as aspiration pneumonia and higher mortality. Various prediction models for aspiration exist, but their clinical utility is limited by methodological heterogeneity.

**Aim:**

This review aimed to evaluate the performance and applicability of these models for stroke patients, informing future model optimization and clinical use.

**Study design:**

A comprehensive search was conducted across nine electronic databases until 20 January 2025. Studies on the development or validation of aspiration risk prediction models in adult stroke patients were included. Data extraction followed the CHARMS checklist, and bias risk and applicability were assessed using the PROBAST tool. The review was prospectively registered with PROSPERO (CRD420251007112).

**Results:**

Eighteen model development studies were included. Most demonstrated good discrimination (AUC/C-index: 0.756–0.955), with 16 showing good applicability. However, all studies had a high risk of bias, mainly due to retrospective designs, small sample sizes (events per variable < 20), inadequate missing data handling, univariate variable selection, and limited external validation. Key predictors included age, NIHSS score, Kubota Water Swallowing Test, history of aspiration, and Glasgow Coma Scale score.

**Conclusion:**

Aspiration risk prediction models for stroke patients show promising predictive performance but are limited by methodological bias and heterogeneity. Future research should prioritize rigorous reporting and multi-center, large-sample external validation to improve model robustness and clinical applicability.

**Systematic review registration:**

PROSPERO CRD420251007112, URL: https://www.crd.york.ac.uk/prospero/display_record.php?ID=CRD420251007112.

## 1 Introduction

Globally, stroke is the second leading cause of death (1) and the foremost cause of disability and death among adults in China (2). The incidence of dysphagia has been reported to affect 42–67% of stroke patients within 3 days of onset, with nearly half of these patients being at a risk of aspiration (3). Aspiration is defined as the entry of food, liquid, saliva, or gastric contents into the airway (larynx, trachea, bronchi, or lungs) during feeding or non-fasting periods (4). Aspiration can precipitate severe complications, such as aspiration pneumonia and asphyxiation, significantly elevating patient mortality and healthcare expenditures (5). Therefore, early identification and the implementation of effective interventions for high-risk individuals are considered crucial strategies for mitigating aspiration risk in stroke patients. Aspiration risk prediction models serve as important tools for quantitatively estimating the probability of aspiration by integrating multidimensional predictors (6), such as demographic characteristics, current and past medical history, clinical assessments, and laboratory indicators. Currently, bedside swallowing screening, video fluoroscopic swallowing study (VFSS), and fiberoptic endoscopic evaluation of swallowing (FEES) are commonly used to assess aspiration risk in patients with stroke (7). However, these approaches may be limited by operational complexity, resource requirements, and feasibility constraints for routine use in all stroke patients, while reliance solely on clinical experience may result in suboptimal accuracy. In recent years, various prediction models for aspiration risk in stroke patients have been developed worldwide. However, substantial heterogeneity exists in predictor selection, modeling methods, and target populations, and their methodological quality and clinical applicability have not been systematically synthesized and evaluated, which may limit their clinical implementation. A comprehensive evaluation of the predictive performance and application value of existing models may facilitate optimization of prediction tools and inform clinical decision-making. Therefore, this study aimed to systematically review aspiration risk prediction models in patients with stroke and to comprehensively analyze their predictive performance, methodological quality, and clinical utility, thereby providing evidence to support model optimization and early identification of high-risk populations in clinical practice.

## 2 Design and methods

### 2.1 Question formulation

The research question for this systematic review was rigorously formulated based on the Population, Index prediction model, Comparator, Outcome, Timing, Setting (PICOTS) framework. The target population consisted of stroke patients, and the index intervention involved aspiration risk prediction models. No specific comparator was identified for this review. The primary outcome measure of interest was the incidence of aspiration in stroke patients, with models intended for application at the time of hospital admission. The relevant study settings included intensive care units, neurology wards, and neurosurgery wards.

### 2.2 Literature search

A comprehensive systematic literature search was executed across several major electronic databases to identify pertinent studies: PubMed, Embase, Web of Science, The Cochrane Library, Cumulative Index to Nursing and Allied Health Literature (ClNAHL), China National Knowledge Infrastructure (CNKI), Wanfang Database, China Science and Technology Journal Database (VIP), and SinoMed. The search encompassed studies published from each database’s inception up to January 2025. This systematic review has been prospectively registered with PROSPERO under registration number CRD420251007112.

The search strategy was meticulously developed, employing a combination of controlled vocabulary [e.g., Medical Subject Headings (MeSH) terms] and free-text terms to ensure broad coverage of relevant literature. For searches conducted in Chinese databases, appropriate Chinese-language subject headings and keywords relevant to stroke, aspiration, and prediction models were utilized. Similarly, for English databases, comprehensive search terms were developed to capture studies on stroke, aspiration, and prediction models. These included MeSH terms and free-text keywords related to various forms of stroke (e.g., “stroke,” “cerebral hemorrhage,” and “brain infarction”), aspiration-related conditions (e.g., “aspiration” and “aspiration pneumonia”), and predictive modeling methodologies (e.g., “predictive value of tests,” “ROC curve,” “nomogram,” “machine learning,” and “prediction model”). The detailed strategies used for the search can be found in the Supplementary material.

### 2.3 Inclusion and exclusion criteria

For the selection of relevant studies, specific inclusion and exclusion criteria were applied. Studies were included if they met the following conditions: (1) the study participants were stroke patients aged 18 years or older; (2) the research presented an original study focusing on the development or validation of aspiration risk prediction models for stroke patients; and (3) the study designs encompassed cohort studies, cross-sectional studies, or case–control studies. Conversely, studies were excluded if: (1) the prediction model incorporated fewer than two predictors; (2) the full text of the study could not be retrieved; or (3) the study was published in languages other than English or Chinese.

### 2.4 Literature screening and data extraction

Literature screening and deduplication were systematically managed using EndNote bibliographic software. Initial screening of studies was performed based on titles and abstracts, followed by a thorough full-text review to confirm eligibility. A standardized data extraction form was specifically developed for this review, guided by the critical appraisal and data extraction for systematic reviews of prediction modelling studies (CHARMS) checklist (8). Data extraction and literature screening were independently conducted by two reviewers. Any discrepancies identified between the reviewers were meticulously resolved through consensus discussion or, when necessary, by arbitration from a third reviewer.

### 2.5 Risk of bias and applicability assessment of included literature

The risk of bias and applicability of the included studies were rigorously assessed using the Prediction Model Risk of Bias Assessment Tool (PROBAST) (9). The PROBAST framework assesses bias across four key domains: participants, predictors, outcome, and analysis, encompassing a total of 20 signaling questions. Similarly, applicability was evaluated across three corresponding domains: participants, predictors, and outcomes. These assessments were independently performed by two reviewers, and findings were meticulously recorded on standardized forms. Each signaling question was judged as ‘Yes,’ ‘Probably yes,’ ‘No,’ ‘Probably no,’ or ‘No information’ based on predefined criteria. The overall risk of bias for each domain was subsequently categorized as ‘Low,’ ‘High,’ or ‘Uncertain.’ Disagreements arising between the reviewers during this evaluation process were resolved through consensus discussion or by consulting a third reviewer.

## 3 Results

### 3.1 Study selection process and results

**Figure 1:**
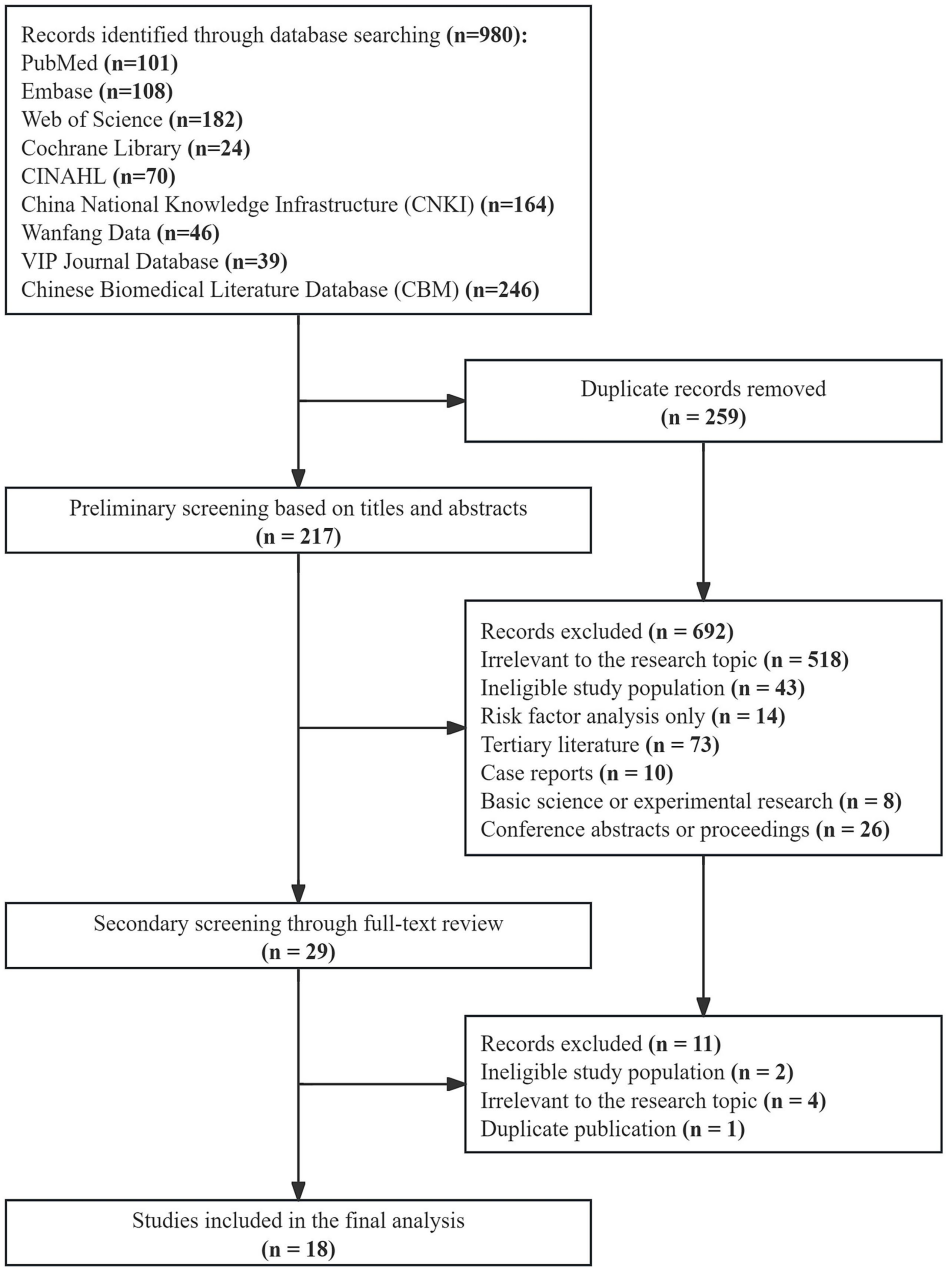
Flow chart of literature screening.

An initial database search yielded 980 articles. Following a rigorous screening process based on the predefined inclusion and exclusion criteria, 18 articles were ultimately included in this systematic review. Of these, 13 studies were published in Chinese, and 5 were published in English. The detailed study selection process, from initial retrieval to final inclusion, is visually depicted in Figure 1.

### 3.2 Characteristics of included studies

All 18 included studies were published within the last 3 years. Of these, 16 originated from China (10–25) and 2 from South Korea (26, 27). Thirteen studies (10, 12–15, 17–24) were published in Chinese, while five (11, 16, 17, 26, 27) were published in English. Regarding study design, only one study (18) employed a cross-sectional design, and one (17) was a prospective cohort study; the remaining studies were retrospective in nature. In terms of outcome definitions, 12 studies (10, 11, 13, 15, 17–20, 22, 23, 26, 27) utilized a comprehensive definition of aspiration as the predicted outcome, whereas 6 studies (12, 14, 16, 17, 21, 24) specifically predicted aspiration pneumonia as a distinct outcome. The fundamental characteristics of the included studies are summarized in Table 1.

**Table 1:** General characteristics of included literature.

Included literature	Publication year	Country	Study type	Study population	Data source	Outcome measured and definition
Zhou et al. (24)	2025	China	Case–control study	Patients with severe stroke	Bozhou City People’s Hospital	Covert aspiration: Food enters the subglottic region or trachea during swallowing, without accompanying cough.
Peng et al. (15)	2022	China	Retrospective cohort study	Patients with severe intracerebral hemorrhage	Neuromedical Intensive Care Unit of a Grade A Tertiary Hospital in Liaoning Province	Aspiration: ① Choking, wheezing, cyanosis, or fluid retention during nasogastric feeding; ② Suctioned fluid suspected to be enteral nutrition formula, with a glucometer reading > 11.1 mmol/L; ③ Gastric pepsin detected in CT scans or secretions.
Xu et al. (20)	2023	China	Case–control study	Stroke patients	Hangzhou Linping District Hospital of Integrated Traditional Chinese and Western Medicine	Aspiration
Lv et al. (14)	2024	China	Retrospective cohort study	Stroke patients	Yongkang Hospital of Traditional Chinese Medicine	Aspiration: ① Clear history of choking; ② Pulmonary inflammatory shadows on chest X-ray/CT; ③ Symptoms of infection such as fever, cough, expectoration, or moist rales; ④ Elevated CRP or procalcitonin.
Cai et al. (10)	2024	China	Retrospective cohort study	Stroke patients with dysphagia undergoing IOE	Second Affiliated Hospital of Wenzhou Medical University	Aspiration
Yu et al. (22)	2024	China	Case–control study	Patients with ischemic stroke	Department of Neurology, Suzhou Ninth Hospital Affiliated with Soochow University	Aspiration: ① Symptoms such as irritative coughing, shortness of breath, cyanosis, or asphyxia; ② Sputum pepsin A > 25 ng/mL; or ③ Presence of symptoms followed by respiratory tract infection, with sputum pepsin A > 25 ng/mL.
Hu et al. (12)	2024	China	Retrospective cohort study	Elderly patients with post-stroke dysphagia after cerebral infarction	Beijing Friendship Hospital Affiliated with Capital Medical University	Aspiration pneumonia: Criteria from “Guidelines for the Diagnosis and Treatment of Community-Acquired Pneumonia.”
Lu (13)	2023	China	Retrospective cohort study	Stroke patients with dysphagia	Rehabilitation Department, Jiawang District People’s Hospital, Xuzhou City	Aspiration
Zhouet al. (23)	2024	China	Case–control study	Patients with acute ischemic stroke	Second Affiliated Hospital of Wenzhou Medical University	Aspiration pneumonia
Xu et al. (21)	2024	China	Retrospective cohort study	Elderly patients with cerebral infarction receiving nasogastric tube feeding	Geriatric Department, Nanjing Central Hospital	Aspiration pneumonia
Wang (18)	2022	China	Cross-sectional study	Stroke patients receiving nasogastric tube feeding	A Grade A Tertiary Hospital in Tianjin	Aspiration: ① Gastric contents overflowing from mouth/nose, accompanied by dyspnea, moist rales, or aspiration pneumonia; ② Gastric contents observed in suctioned airway material or secretions.
Wang et al. (17)	2024	China	Retrospective cohort study	Patients with post-stroke dysphagia	First Affiliated Hospital of Zhengzhou University	Aspiration pneumonia: Criteria from “Expert Consensus on the Diagnosis and Treatment of Adult Aspiration Pneumonia.”
Xie (19)	2024	China	Retrospective cohort study	Patients with acute cerebral infarction	Jingzhou Fifth People’s Hospital	Aspiration: ① Rapid breathing, choking, increased heart rate; ② Enteral nutrition formula observed in mouth/nose or sputum; ③ Respiratory secretions pH < 7; ④ Positive for gastric pepsin; ⑤ Gastric contents visible via bronchoscope.
Ryu et al. (27)	2024	South Korea	Retrospective cohort study	Patients with post-stroke dysphagia (PSD)	Dankook University Hospital, South Korea	Aspiration: Clearly observed aspiration phenomenon during Videofluoroscopic Swallowing Study (VFSS).
Park et al. (26)	2023	South Korea	Retrospective cohort study	Patients with acute stroke	Pohang Stroke and Spine Hospital, South Korea	Aspiration: At least one swallow detected by VFSS during hospitalization, with a Penetration-Aspiration Scale (PAS) score of 6–8.
Chen et al. (11)	2024	China	Retrospective cohort study	Patients with post-stroke dysphagia (PSD)	Departments of Neurology, Neurosurgery, and Rehabilitation, Guizhou Provincial People’s Hospital	Aspiration: ① Positive Kubota Water Swallowing Test; or ② Coughing, rapid breathing, cyanosis, increased heart rate, difficulty speaking, or hoarseness during eating; or ③ Bronchoscopy: presence of oral contents, food particles, gastric pepsin, or bile.
Wang et al. (25)	2024	China	Prospective cohort study	Patients with acute ischemic stroke	Affiliated Hospital of Jiangnan University	Aspiration: ① Irritative coughing; or ② Abnormal phonation; or ③ Cyanosis, dyspnea, decreased oxygen saturation; or ④ Gastric contents in the airway; or ⑤ Food entering below the vocal cords during Fiberoptic Endoscopic Evaluation of Swallowing (FEES) examination.
Wang et al. (16)	2023	China	Retrospective cohort study	Patients with acute ischemic stroke	Jinshan Hospital Affiliated with Fudan University	Aspiration pneumonia: Diagnostic criteria from the ATS/IDSA CAP guidelines.

IOE. intermittent oral-to-esophageal feeding; PSD, post-stroke dysphagia; VFSS, videofluoroscopic swallowing study; PAS, penetration-aspiration scale.

### 3.3 Characteristics of included prediction models

All 18 included studies were dedicated to model development. The sample sizes utilized for model construction ranged from 60 to 2,556 participants, with the number of events per variable (EPV) ranging from 1.318 to 24.889. Logistic regression modeling was employed in 16 studies (10–24). One study (26) integrated multiple machine learning methods, such as regularized logistic regression, random forest, extreme gradient boosting, support vector machine, K-nearest neighbors, and Naive Bayes. Additionally, deep learning methods were utilized in one study (27). Regarding the handling of missing data, most studies did not report the presence or management of missing data; only one study (26) employed multiple imputation, while another (27) directly excluded cases with missing data. In terms of predictor variables, the number of candidate predictors considered ranged from 3 to 35. Continuous variables were appropriately handled in three studies (13, 21, 23). Furthermore, the sole reliance on univariate analysis for model construction was avoided in seven studies (11, 12, 16–18, 21, 26). These characteristics are summarized in Table 2.

**Table 2:** General characteristics of included predictive models.

Included literature	Candidate predictors	Missing data	Sample size (cases, development/validation)	Number of outcome events (cases, development/validation)	Events per variable (EPV)	Model development
	Number	Categorization of continuous variables	Number of cases	Handling method		Modeling method
Zhou et al (23)	8	All converted	–	–	60/–	28/–
Peng et al. (15)	15	Partially retained	–	–	368/53	147/–
Xu et al. (20)	11	Partially retained	–	–	110/–	55/–
Lv et al. (14)	16	Partially retained	–	–	255/–	55/–
Cai et al. (10)	22	Partially retained	–	–	146/–	29/–
Yu et al. (22)	15	Partially retained	–	–	316/–	89/–
Hu et al. (12)	20	Partially retained	–	–	196/84	84/37
Lu (13)	12	All converted	–	–	215/–	59/–
Zhou et al. (23)	35	Partially retained	–	–	452/–	119/–
Xu et al. (21)	20	All converted	–	–	155/–	71/–
Wang (18)	34	Partially retained	–	–	366/155	96/53
Wang et al. (17)	17	Partially retained	–	–	148/–	46/–
Xie (19)	16	Partially retained	–	–	200/50	42/14
Ryu et al. (27)	3	Retained as continuous	24	Exclusion	85	35
Park et al. (26)	18	Partially retained	Yes (1)	Multiple imputation	2556/852	448
Chen et al. (11)	16	Partially retained	–	–	330/82	147/37
Wang et al. (25)	30	Partially retained	–	–	359/64, 105	61/15, 13
Wang et al. (16)	20	Partially retained	–	–	2281/977	220/87

LR. logistic regression; ANN, artificial neural network; RLRs, regularized logistic regressions; RF, random forest; XGB, extreme gradient boosting; SVM, support vector machine; KNN, *k*-nearest neighbors algorithm; NB, Naive Bayes.

### 3.4 Performance and predictors of included prediction models

The area under the receiver operating characteristic curve (AUC) and C-statistic (C-index) values reported in 16 studies (10–12, 14–22, 24, 26, 27) ranged from 0.756 to 0.955, indicating good discriminatory ability of the models. Calibration was reported in 12 studies (11–20, 22, 24), with 3 of these (18, 19, 24) exclusively employing the Hosmer–Lemeshow test. Model clinical utility was assessed using decision curve analysis (DCA) in seven studies (11, 12, 14, 16–18, 22). Regarding validation methods, internal validation techniques, such as random splitting, K-fold cross-validation, and Bootstrap resampling, were employed in nine studies (11, 15–18, 20, 22, 24, 26). External validation was conducted in six studies (11, 12, 15, 17–19). Concerning model presentation, model equations were derived based on logistic regression coefficients in two studies (13, 19). Nomograms were used for presentation in 12 studies (10–12, 14–18, 20, 22, 26), while risk scores were presented in 3 studies (21, 23, 24). One study (27) did not specify the model presentation format. The final models incorporated between 3 and 13 independent predictor variables. The five most frequently reported predictors were age (*n* = 10), NIHSS score (*n* = 9), Kubota Water Swallowing Test (*n* = 4), history of aspiration (*n* = 4), and Glasgow Coma Scale (GCS) score (*n* = 4). These findings are detailed in Table 3.

**Table 3:** Performance and predictive factors of included predictive models.

Included literature	Model performance	Validation method	Model presentation	Predictive factors
	Diagnosis (AUC) development/validation	Calibration	Clinical utility	Internal/external
Zhou et al. (23)	–	–	–	–/–
Peng et al. (15)	0.955/0.891	Calibration curve	–	Internal validation (1) and external validation
Xu et al. (20)	0.885, C-index 0.831/–	Calibration curve, H-L test	–	Bootstrap resampling/–
Lv et al. (14)	C-index 0.891/–	Calibration curve	DCA	–/–
Cai et al. (10)	0.756, C-index 0.705/–	Calibration curve, H-L test	–	–/–
Yu et al. (22)	0.872/0.859	Calibration curve	DCA	Random split validation/–
Hu et al. (12)	−/0.957	Calibration curve	DCA	–/Random split validation
Lu (13)	–/–	–	–	–/–
Zhou et al. (23)	0.831/internal 0.827	H-L test	–	Bootstrap resampling/–
Xu et al. (21)	0.823/–	–	–	–/–
Wang (18)	0.904/0.847	H-L test	DCA	10-fold cross-validation/external validation
Wang et al. (17)	0.902/–	–	–	–/–
Xie (19)	0.812/0.885	H-L test	-	–/Random split validation
Ryu et al. (27)	Vmax 0.715 (2)	–	–	–
Park et al. (26)	0.810 (3)/–	–	–	Random split validation and 5-fold cross-validation
Chen et al. (11)	0.834/0.882	Calibration curve, H-L test	DCA	Bootstrapping, bootstrap/external validation
Wang et al. (25)	0.853/0.872, 0.877	Calibration curve, H-L test	DCA	Resampling/spatial validation
Wang et al. (16)	C-index 0.872/C-index 0.847	Calibration curve	DCA	3-fold cross-validation, bootstrap resampling/external validation

NIHSS, National Institutes of Health Stroke Scale; VDS, Verbal Description Scale; SSA, Standard Swallowing Assessment; GCS, Glasgow Coma Scale; Hcy, homocysteine; CRP, C-reactive protein; NLR, neutrophil-to-lymphocyte ratio; LER, leukocyte-to-erythrocyte ratio; BMI, body mass index.

1) Specific validation methods not reported; 2) Vmax performance; 3) Ridge regression model.

### 3.5 Risk of bias and applicability assessment of included literature

All 18 included studies were assessed as having a high risk of bias. However, 16 of these studies (10, 11, 13–20, 22–24, 26, 27) were deemed to have good applicability. The comprehensive results are summarized in Table 3.

#### 3.5.1 Bias risk assessment results

In the participants’ domain, 16 studies (10–17, 19–24, 26, 27) exhibited a high risk of bias. This was primarily due to their reliance on existing data for retrospective case analysis, which could lead to prediction results deviating from true clinical scenarios. In the predictors domain, 11 studies (10, 12–14, 17, 20, 21, 23, 24, 26, 27) were retrospective and did not clearly state whether predictors were assessed without knowledge of the outcome. Among these, five studies (10, 14, 21, 23, 24) did not provide detailed descriptions of the predictor measurement methods, leading to an “unclear” risk of bias assessment for this domain. In the outcome domain, one study (14) was rated as having a “high risk” of bias due to potential incorporation bias, where candidate predictors overlapped with the definition of the aspiration outcome. Additionally, five studies (10, 13, 20, 21, 24) did not clearly define the clinical outcome, resulting in an “unclear” rating, while the remaining studies in this domain were at low risk of bias.

A high risk of bias was identified across all included studies in the analysis domain due to several common issues: (1) Except one study (26), the sample sizes in other studies were insufficient [events per variable (EPV) < 20], and no parameter adjustments were reported, potentially leading to underfitting or overfitting of the models. (2) In three studies (13, 21, 23), continuous variables were transformed into categorical variables, but no specific justification was provided for the threshold cut-off points used for categorization, which may have resulted in a loss of information. (3) Sixteen studies (10–24) did not report on missing data, and one study (27) employed complete case analysis, potentially introducing selection bias and overestimating model discriminatory ability. (4) Variable selection in 11 studies (10, 13–15, 17, 19, 20, 22–24, 27) was based solely on univariate analysis, failing to consider inter-variable interactions, which could lead to the omission of key predictors. (5) Six studies (13, 17, 21, 23, 26, 27) did not provide an assessment of model calibration, and three studies (18, 19, 24) only reported the Hosmer–Lemeshow goodness-of-fit test, indicating incomplete calibration information. (6) Finally, 9 studies (10, 12–14, 19, 21, 23, 27) did not perform internal validation, which could result in overly optimistic model performance estimates.

#### 3.5.2 Applicability assessment results

Of the 18 included studies, 2 (12, 21) were assessed as having a high applicability risk due to their exclusive inclusion of patients older than 65 years. The remaining studies exhibited a low overall applicability risk. These findings are presented in Table 4.

**Table 4:** Risk of bias and applicability assessment of included studies.

Included literature	Risk of bias	Applicability	Overall
	Participants	Predictors	Outcome
Zhou et al. (23)	High	Unclear	Low
Peng et al. (15)	High	Low	Low
Xu et al. (20)	High	Unclear	Unclear
Lv et al. (14)	High	Unclear	High
Cai et al. (10)	High	Unclear	Unclear
Yu et al. (22)	High	Low	Low
Hu et al. (12)	High	Unclear	Low
Lu (13)	High	Unclear	Unclear
Zhou et al. (23)	High	Unclear	Unclear
Xu et al. (21)	High	Unclear	Unclear
Wang (18)	Low	Low	Low
Wang et al. (17)	High	Unclear	Low
Xie (19)	High	Low	Low
Ryu et al. (27)	High	Unclear	Low
Park et al. (26)	High	Unclear	Low
Chen et al. (11)	High	Low	Low
Wang et al. (25)	Low	Low	Low
Wang et al. (16)	High	Low	Low

## 4 Discussion

### 4.1 High risk of bias in aspiration risk prediction models for stroke patients

Sixteen of the included studies (10–24) originated from China and were published within the last 3 years, indicating a growing focus on aspiration risk prediction models for stroke patients in China. However, all 18 included studies exhibited a high risk of bias, primarily attributable to methodological shortcomings that compromised their scientific rigor and robustness. The specific reasons are detailed as follows: First, most studies relied on retrospective data. While such data are readily accessible and cost-effective, their use is prone to selection and information biases, potentially leading to an overestimation of risk association strengths. Second, the majority of studies reported an events per variable (EPV) of less than 20. This inadequacy not only diminishes the model’s capacity to capture complex clinical relationships but also increases the likelihood of model overfitting or underfitting, thereby compromising its stability and accuracy (28). Third, conventional logistic regression methods were employed for model construction in 16 studies (10–24), with only 2 studies (26, 27) exploring machine learning or deep learning approaches. While logistic regression is widely utilized in clinical practice, it presents inherent limitations in addressing non-linear relationships and variable interactions. Conversely, machine learning methods offer the potential to enhance predictive performance through feature engineering. Moreover, most studies failed to report on missing data, and only two studies (26, 27) described their handling of missing data. Improper management of missing data can introduce selection bias and lead to information loss, consequently overestimating model discrimination. Future research is encouraged to utilize techniques such as multiple imputation or Bayesian models for missing data management, thereby ensuring data completeness and model reliability. Finally, external validation was conducted in only six studies (11, 12, 15, 17–19), and these validations were limited to healthcare institutions of similar tiers or comparable populations. This limitation restricts the generalizability and transportability of the models across diverse geographical regions and patient populations (29). In summary, current models are still characterized by notable methodological limitations, particularly in study design rigor, sample size adequacy, missing data management, and external validation. Future studies should place greater emphasis on methodological standardization during model development and reporting to improve the scientific validity and reliability of prediction models.

### 4.2 Aspiration predictors in stroke patients exhibit good clinical effectiveness

Early identification and intervention for high-risk factors in stroke patients are paramount for reducing the incidence of aspiration. This study identified age, NIHSS score, Kubota Water Swallowing Test, history of aspiration, and GCS score as the primary predictors of aspiration in stroke patients, consistent with findings from other studies (30). These factors not only possess biological plausibility but have also demonstrated clinical effectiveness, as validated by multiple studies. This suggests that clinical nurses should emphasize the assessment and prevention related to these characteristic variables. Age, identified as an independent risk factor for aspiration, is likely associated with diminished swallowing reflex, decreased coughing ability, and an increased burden of comorbidities in older patients. The NIHSS score, serving as an assessment tool for neurological deficits, is closely related to dysphagia. A study by Kumar et al. (31) involving 323 patients with acute ischemic stroke indicated that an NIHSS score of ≥12 was a significant predictor of persistent dysphagia, suggesting a higher aspiration risk for patients with elevated NIHSS scores. The Kubota Water Swallowing Test is widely recommended due to its simplicity and high sensitivity. However, its relatively low specificity may lead to an elevated false-positive rate; thus, modified versions of the Kubota Water Swallowing Test are frequently employed in clinical practice (32). Furthermore, international expert consensus recognizes a history of aspiration as a critical risk factor for aspiration in critically ill patients (33) because it reflects the previous impaired swallowing function. If this impaired state is not improved, it will continue to increase the risk of overt aspiration again (34). Additionally, a reduced GCS score is also considered a risk factor for aspiration, as it indicates impaired consciousness, which can suppress the cough reflex and pharyngeal sensory function, thereby increasing the risk of aspiration (35). Despite the demonstrated clinical effectiveness of these factors, their combined application necessitates careful consideration of clinical specificity and practicality. Currently, most studies primarily rely on clinical scales, with an evident lack of integrated analysis incorporating biomarkers and imaging features. Therefore, future research is advised to continue exploring the interactive effects of predictor variables and to construct multi-dimensional prediction systems, thereby optimizing model accuracy and utility to provide a scientific basis for individualized aspiration prevention strategies.

### 4.3 Sources of model heterogeneity and future optimization directions

Current prediction models for aspiration risk in patients with stroke exhibit substantial heterogeneity in modeling approaches, outcome definitions, and study designs, which may contribute to variability in model performance and identified predictors. Most studies employed traditional logistic regression, whereas only a few applied machine learning techniques. Current evidence does not demonstrate a consistent advantage of advanced algorithms in predictive performance. Differences in feature selection procedures and model development strategies, together with limited sample sizes and insufficient external validation, further increase methodological heterogeneity and make direct comparisons across models difficult. Variability in outcome definitions also represents a major source of heterogeneity. In this review, the included studies predicted either aspiration or aspiration pneumonia. Aspiration reflects swallowing dysfunction and neurological impairment, whereas aspiration pneumonia is a secondary infectious complication influenced by additional factors such as inflammatory response and comorbidities. Consequently, models predicting aspiration more frequently incorporated neurological severity and swallowing-related variables, whereas those predicting aspiration pneumonia tended to include inflammatory markers and comorbidity-related factors. Inconsistent endpoint definitions, particularly the limited differentiation between symptomatic and asymptomatic aspiration, may therefore substantially influence predictor selection and model structure. Furthermore, most included studies adopted single-center retrospective designs, and high-quality prospective studies with rigorous external validation remain limited. The geographical distribution of the evidence was also highly concentrated, with most studies conducted in China and only a small number from other countries. Such concentration may limit the generalizability of existing models across different healthcare systems and patient populations. Future research should prioritize standardized outcome definitions, multicenter and multinational collaboration, and robust external validation, while optimizing modeling strategies under the premise of maintaining clinical interpretability, to enhance model robustness and cross-population applicability.

## 5 Conclusion

This study systematically evaluated 18 included articles, revealing that existing aspiration risk prediction models for stroke patients generally demonstrate good predictive performance and applicability but are commonly associated with a high risk of bias. Furthermore, due to significant heterogeneity in model construction methods, predictor combinations, and outcome definitions, quantitative data synthesis was not conducted in this study. Consequently, the conclusions are based on qualitative analysis, which may limit the strength of the evidence. Future research should rigorously adhere to the Transparent Reporting of a Multivariable Prediction Model for Individual Prognosis or Diagnosis (TRIPOD) statement to standardize study design and reporting. These findings may support early risk stratification of aspiration in stroke patients and provide a reference for future model development and clinical implementation.

## Data Availability

The original contributions presented in the study are included in the article/Supplementary material, and further inquiries can be directed to the corresponding author.
