# Supplementary material for: Prediction models for aspiration risk in stroke patients: a systematic review
Source: Front Neurol. 2026 Apr 16;17:1700285. doi: 10.3389/fneur.2026.1700285 (PMC13170933; doi:10.3389/fneur.2026.1700285)
Supplement: Supplementary file 1 [file Table_1.docx]

**Table S1.** List of final predictor variables included in each of the 18 prediction models for post-stroke aspiration

| **Included Literature** | **Number of Predictors** | **Specific Predictors** |
| --- | --- | --- |
| Zhou Pengfei et al.^8^ | 4 | Age, Consciousness disorder, Swallowing dysfunction, Gastrointestinal dysmotility |
| Peng Yu et al.^9^ | 5 | Nasogastric tube size, Gastric residual volume, History of aspiration, NIHSS score, Kubota Water Swallowing Test |
| Xu Jinjuan et al.^10^ | 5 | Age, Site of infarction, Lesion location, Hypertension, NIHSS score |
| Lv Wenyi et al.^11^ | 5 | NIHSS score, Verbal Description Scale (VDS) score, Standardized Swallowing Assessment (SSA) score, Nasogastric feeding, Gastroesophageal reflux |
| Cai Xiaotiao et al.^12^ | 8 | History of stroke, History of aspiration, Location of stroke, Voluntary cough, Kubota Water Swallowing Test, NIHSS score, Tube feeding position, Depth of tube insertion |
| Yu Lingying et al^13^ | 6 | Age, NIHSS score, Multiple lesions, Homocysteine (Hcy) level, Voluntary cough, Kubota Water Swallowing Test |
| Hu Jieqiong et al.^14^ | 7 | Age, Underlying pulmonary disease, NIHSS score, Improper positioning, Electrical stimulation therapy, Pharyngeal residue, Peripheral blood levels of CRP/NLR/LER |
| Lu Qianqian15 | 6 | Age, Type of stroke, Location of stroke, Mechanical ventilation, Food texture, Feeding posture |
| Zhou Xiaoming et al.^16^ | 6 | Age, GCS score, History of stroke, History of diabetes, Dysphagia, Hypoalbuminemia |
| Xu Min et al.^17^ | 7 | Number of intubations, Daily nasogastric feeding volume, Swallowing function training, Voluntary cough, Vomiting, Sedative-hypnotic drugs, GCS score |
| Wang Yuxin^18^ | 9 | History of aspiration, Length of hospital stay, Depth of tube insertion, Mechanical ventilation, Nebulization therapy, Vomiting, GCS score, White blood cell count, Platelet count |
| Wang Tianmeng et al.^19^ | 5 | Age, Barthel Index, Kubota Water Swallowing Test, Nutritional support method, Neutrophil-to-Lymphocyte Ratio (NLR) |
| Xie Xiaolei^20^ | 6 | Nasogastric tube size, Depth of tube insertion, Gastric residual volume, History of aspiration, NIHSS score, GCS score |
| Ryu et al.^24^ | 3 | Hyoid bone movement distance (Horizontal, Vertical, Diagonal) |
| Park et al.^25^ | 13 | Age, Male sex, Previous cerebrovascular event, Posterior circulation stroke, Altered mental status, Aphasia, modified Rankin Scale (mRS) score, Initial systolic BP, Days to VFSS, Facial symmetry, BMI, Left-sided lesion, Diastolic BP |
| Chen et al.^21^ | 7 | Age, Sex, Feeding mode, Dysarthria, NIHSS score, Abnormal cough, Facial palsy |
| Wang et al.^22^ | 4 | Sputum suction, Brainstem infarction, Temporal lobe infarction, Barthel Index score |
| Wang et al.^23^ | 11 | Age, NIHSS score, Dysphagia, Atrial fibrillation, Heart failure, Renal insufficiency, Hepatic insufficiency, Fasting blood glucose, C-reactive protein (CRP), Neutrophil percentage, Prealbumin |
